# Empowering community health workers in rural Ethiopia with blended learning: an offline mobile application to enhance trainings and healthcare services

**DOI:** 10.1093/oodh/oqaf022

**Published:** 2025-09-09

**Authors:** Etsegent Arega Asmamaw, Mark Ryan Begley, Temesgen Ayehu Bele, Ruth Diriba Debar, Tamene Feyissa Egnuni, Julie Anne Krause, Abraham Zerihun Megenetta, Israel Ataro Otoro, Tibebu Benyam Sado, Netsanet Fetene Wendmagegn

**Affiliations:** MERL Office, Ethiopia Country Program, Last Mile Health, AG Grace Building, 8th Floor, Djibouti Road, Bole Sub-City, Addis Ababa, Ethiopia; Global MERL Office, Last Mile Health, 68 Harrison Ave Ste 605 PMB 31738, Boston 02111-1929, Massachusetts; Program Office, Ethiopia Country Program, Last Mile Health, AG Grace Building, 8th Floor, Djibouti Road, Bole Sub-City, Addis Ababa, Ethiopia; Program Office, Ethiopia Country Program, Last Mile Health, AG Grace Building, 8th Floor, Djibouti Road, Bole Sub-City, Addis Ababa, Ethiopia; Program Office, Ethiopia Country Program, Last Mile Health, AG Grace Building, 8th Floor, Djibouti Road, Bole Sub-City, Addis Ababa, Ethiopia; Global MERL Office, Last Mile Health, 68 Harrison Ave Ste 605 PMB 31738, Boston 02111-1929, Massachusetts; Country Director Office, Ethiopia Country Program, Last Mile Health, AG Grace Building, 8th Floor, Djibouti Road, Bole Sub-City, Addis Ababa, Ethiopia; Primary Health Care and Community Engagement Lead Executive Office, Ministry of Health, 1234 Sudan Street, Arada Sub-City, Addis Ababa, Ethiopia; Program Office, Ethiopia Country Program, Last Mile Health, AG Grace Building, 8th Floor, Djibouti Road, Bole Sub-City, Addis Ababa, Ethiopia; MERL Office, Ethiopia Country Program, Last Mile Health, AG Grace Building, 8th Floor, Djibouti Road, Bole Sub-City, Addis Ababa, Ethiopia

**Keywords:** health extension workers, offline mobile application, training, rural healthcare, localization, blended learning, Ethiopia

## Abstract

**Introduction:**

This study addresses challenges in delivering in-service Integrated Refresher Training to 40 000 Health Extension Workers (HEWs) in rural Ethiopia. Through an offline multilingual mobile application, Extension Essentials (EE), it aims to improve their knowledge and skills through a less costly blended learning approach combining in-person facilitation with offline digital self-learning.

**Methods:**

In a pilot study from November 2021 to May 2022, a mixed-methods evaluation assessed knowledge, skills, satisfaction and cost-effectiveness of training focused on Reproductive, Maternal, Newborn and Child Health. HEWs and their supervisors were allocated by district into two groups: one receiving only conventional in-person training (conventional Integrated Refresher Training [IRT] control group) and the other using the mobile application for blended training (blended IRT treatment group). The evaluation utilized a quasi-experimental before-after matched comparison group design with 20 districts in the blended IRT treatment group study arm and 20 districts in the conventional IRT control group study arm. The blended IRT treatment group and conventional IRT control group sites were selected in consultation with the Ministry of Health using convenience sampling and matched based on location, population size and health infrastructure.

**Findings:**

Data showed learners engaged with the mobile app for an average of 2.8 hours per day (more than expected 2 hours) during self-learning, with high completion rates for activities (95%) and quizzes (88%). Learner knowledge scores improved significantly more from pre- to post-training in the blended IRT treatment group as compared to the conventional IRT control group (adjusted difference-in-differences: 2.01 percentage points, *P* = 0.001; 95% CI: 0.8–3.2), though the difference was not programmatically meaningful, indicating that both training modalities were similarly effective at improving knowledge. Skills assessment scores improved significantly in the blended IRT treatment group from 60% pre-training to 90% post-training (*P* < 0.001). There was no skill assessment conducted for the conventional IRT control group. Additionally, the blended approach reduced recurring training costs by 39% as compared to the conventional training.

**Conclusion:**

Findings indicate that the EE effectively enhances training through a flexible, user-friendly platform that addresses connectivity barriers and costs less than traditional methods, while yielding similar knowledge outcomes. Blended learning solutions are vital for scaling healthcare training in remote settings, warranting research on long-term impacts and broader applicability.

## INTRODUCTION

Ethiopia’s Health Extension Program (HEP) is widely recognized as a global model for expanding access to primary healthcare in rural and remote areas. The program employs over 40 000 Health Extension Workers (HEWs), predominantly women, who deliver preventive, promotive and curative health services to their communities. Over the past two decades, HEWs have played a pivotal role in reducing child and maternal mortality rates in Ethiopia [1]. HEWs in Ethiopia play a crucial role in delivering essential healthcare services, particularly in rural and underserved areas. However, maintaining and enhancing their competencies requires continuous training and support [2]. The Ethiopian Ministry of Health (MOH) has implemented Integrated Refresher Training (IRT) programs to address this need, but challenges such as geographic dispersion, cost constraints and limited connectivity have hindered their effectiveness [3, 4]. Previous research has shown that mobile learning interventions can enhance access to educational materials, improve knowledge retention and facilitate flexible learning for healthcare workers in similar contexts [5, 6].

Despite these achievements, the program faces ongoing challenges, particularly in maintaining the quality of services and ensuring that HEWs have access to high-quality, up-to-date training. Continuous professional development is essential for HEWs to effectively address the evolving health needs of their communities, including reproductive, maternal, newborn and child health (RMNCH), non-communicable diseases (NCDs) and communicable diseases [7, 8].

To address these challenges, the MOH has implemented IRT for HEWs, which are conducted every 2 years. These face-to-face trainings cover six key modules: RMNCH, hygiene and environmental health, NCDs, major communicable diseases, social behavior change communication and first aid/emergency care. However, the conventional IRT model has several limitations, including ineffective learner engagement, limited interactive content and high resource intensity due to the extended duration of in-person sessions. These challenges have hindered the ability of HEWs to fully benefit from the training, ultimately impacting the quality of care they provide [9, 10].

Effective in-service training is critical for the continuous professional development of frontline healthcare workers, particularly in low-resource settings. Traditional face-to-face training programs often face logistical and financial challenges, leading to inconsistent participation and limited scalability. The advent of digital learning technologies has provided new opportunities to overcome these barriers, particularly through blended learning approaches that integrate digital and in-person training components [11, 12].

Blended learning combines face-to-face instruction with digital and mobile learning solutions, offering an effective approach to addressing training challenges in resource-constrained settings [13, 14]. Research in LMICs suggests that blended learning can enhance knowledge retention, provide flexibility for learners and reduce training costs while maintaining the quality of learning outcomes [15, 16]. Additionally, integrating digital tools into training programs has been found to support self-paced learning and enable continuous professional development among community health workers [17, 18].

In response to these limitations, the MOH, in collaboration with Last Mile Health (LMH), has pioneered a blended learning approach to revolutionize HEW training. This innovative model combines digital and in-person components to enhance the effectiveness and efficiency of IRT, particularly for the RMNCH module. The blended learning approach leverages digital technology, including mobile applications, multimedia content and real-time data analytics, to create a more engaging and accessible training experience. By reducing the number of in-person training days, the blended model also addresses logistical and financial constraints, making it a more sustainable solution for scaling up HEW training across Ethiopia.

The blended learning approach is grounded in adult learning principles and incorporates a variety of instructional design features, such as role-plays, case studies, demonstrations and group discussions. The digital component of the training is delivered through the Extension Essentials (EE) app, which provides HEWs with access to multimedia resources, including animated videos, lecture videos and illustrations, all available in local languages. The app also includes interactive quizzes and continuous assessments, allowing HEWs to track their progress and identify areas for improvement. Importantly, the app can be used offline, ensuring that HEWs in remote areas with limited internet connectivity can still access the training materials [19].

This pilot initiative represents a significant step forward in addressing the challenges of traditional IRT models. By integrating digital tools into HEW training, the blended learning approach not only enhances the quality of training but also provides a scalable and cost-effective solution for improving community health outcomes. This manuscript presents the findings from the pilot implementation of the blended RMNCH IRT, highlighting its impact on HEW knowledge, skills and cost reduction in training HEWs, as well as its potential for broader adoption across Ethiopia’s health system.

Blended learning, which combines conventional face-to-face instruction with digital self-learning tools, has been increasingly adopted in global health education [20]. Studies have demonstrated that such approaches can lead to comparable or superior learning outcomes compared to traditional methods while being more cost-effective and scalable [21, 22]. For example, a study in Ghana found that community health nurses trained through a blended learning approach demonstrated improved knowledge retention and clinical decision-making skills compared to those who underwent traditional training [23]. Similarly, research in India and Kenya highlighted that blended learning strategies enhance the confidence and competency of community health workers while overcoming logistical barriers associated with in-person training [24, 25].

In Ethiopia, recent mobile-based training initiatives, including the use of offline multilingual applications, have shown promise in overcoming internet connectivity barriers and ensuring that HEWs can access training materials anytime and anywhere [26, 27]. The EE mobile application represents an innovative effort to address the limitations of conventional in-service training by providing offline, multilingual digital learning content for HEWs. By integrating digital learning with in-person facilitation, EE aims to enhance HEWs’ competencies in RMNCH while ensuring cost-effectiveness and ease of implementation. This study builds on existing evidence supporting blended learning approaches and contributes new insights into their application in rural healthcare training programs in Ethiopia.

## METHODS

A mixed-method evaluation was conducted to assess the effectiveness of a pilot program providing in-service refresher training to HEWs using blended (partially digital) training in comparison to conventional IRT control group (fully in-person) training. Impact was compared using a quasi-experimental before-after matched comparison group design. The pilot was conducted in 20 blended IRT treatment group districts and 20 conventional IRT control group districts across Oromia, Sidama, SNNPR and Amhara regions. Blended learning training sites were selected with the MOH using convenience sampling, and conventional training sites were selected and matched based on location, population size and health infrastructure including number of health centers, health posts and HEWs.

Training approach: The conventional IRT is a fully in-person, seven-day training conducted at the district or zonal level without the use of digital technology. Its primary focus is on knowledge acquisition, and it does not include any formal assessment of practical skills.

In contrast, the blended learning approach combines 4 days of in-person instruction with 5 days of self-paced digital learning (~2 hours per day) delivered through the EE app. This modality was designed to improve the effectiveness and efficiency of training by integrating multimedia content, continuous assessments and real-time data for quality improvement. The blended training emphasizes both knowledge and skill development, aligns with MOH-accredited Continuous Professional Development (CPD) centers and actively involves HEW supervisors to provide ongoing post-training support.

Evaluation Framework: To evaluate the blended training pilot, evaluation questions were aligned with the Kirkpatrick Model which measures four levels of outcomes: (i) reaction, or the degree to which participants find the training favorable, engaging and relevant to their jobs; (ii) learning, or the degree to which participants acquire the intended knowledge, skills, attitudes, confidence and commitment based on their participation in the training; (iii) behavior, or the degree to which participants apply what they learned during training when they are back on the job; and (iv) results, or the degree to which targeted outcomes occur as a result of the training, support and accountability package [28]. Due to the short timeframe of the study (7 months, November 2021 to May 2022), the research focused on measuring the first and second level of outcomes: reaction and learning. The third and fourth levels (behavior and results) focus on mid- and long-term outcomes, which take more time to manifest and are influenced by many contextual factors [29]. Table 1 shows Blended RMNCH In-Service Training pilot evaluation aims and questions.

**Table 1:** Blended RMNCH In-service training pilot evaluation aims and questions.

**Evaluation Aims**	**Evaluation Questions**
**Aim 1: Reach** To measure the overall reach of the blended RMNCH IRT and determine learner characteristics and reach.	How many learners participate in and complete the RMNCH Blended In-Service Training? What are the demographic characteristics of the learners (e.g. age, gender, education level, etc.)?
**Aim 2: Reaction** To understand how the blended RMNCH IRT fulfills learners’ needs and addresses gaps in conventional IRT implementation.	To what extent do learners engage in the program? Are there any differences in completion of the in-person and digital self-learning components? What components of the course are the most relevant and valuable to the learner?
**Aim 3: Learning** To determine if the blended RMNCH IRT supports increased knowledge, self-efficacy and skills in key RMNCH competencies needed for community health work.	What level of competency do learners have in key course concepts before and after their participation in in-service training? How much confidence do learners have in their abilities to apply this knowledge and these skills in their work before and after their participation in in-service training?
**Aim 4: Application** To determine how learners apply key RMNCH knowledge and skills within their work.	How do learners actually apply knowledge and skills from in-service training? How do HEW Supervisors use in-service training data in follow-up supervision visits?
**Aim 5: Results** To determine to what extent the blended IRT approach contributes to community health systems improvements (specifically the cost of implementing quality IRT).	What differences exist in the quality, efficiency and effectiveness of RMNCH Blended In-Service Training between conventional in-person training methodologies and the blended approach? What differences exist in the costs of delivering RMNCH Blended In-Service Training in both conventional and blended training modalities?

Ethical approval was obtained from Ethiopia Public Health Association and informed consent was secured from all participants prior to their involvement in the study and data were collected from November 2021 to May 2022. Assessment areas and methods are outlined in Table 2.

**Table 2:** Assessment areas and methods.

**Method**	**Assessment Area**	**Timing and Study Arm**
**Learner surveys**	Learner characteristics ICT competence RMNCH knowledge Satisfaction with the training Usability of digital platform^*^ Feasibility of blended approach at scale^*^ Challenges and suggested improvements^*^	Pre- and post-training Blended and conventional IRT control group * measured only at blended training sites
**Passive in-app activity data**	Engagement with digital components Implementation fidelity to blended approach	Continuous Blended learners only
**Facilitator surveys**	Satisfaction with the curriculum design, digital platform Quality of facilitation Learner performance Challenges and suggested improvements Feasibility of blended approach at scale	Post-training Blended learners only
**Supervisor surveys**	Ability to support HEWs during blended IRT Satisfaction with the blended learning IRT Use of training data in follow-up supervision visits Challenges and suggested improvements	Post-training Blended learners only
**Skills assessments**	RMNCH skills	Pre- and post-training Blended learners only
**Learner focus group discussions**	Early application of RMNCH knowledge and skills Experience with the digital platform Satisfaction with the instructional design, course content Use of digital training components after IRT Opinion of blended training vs. in-person training Feasibility of blended approach at scale Role of supervisor in blended IRT	Post-training Blended learners only
**Cost data capture**	Cost per learner of delivering standard and blended IRT for the RMNCH module Cost per training output and outcome indicators	Training period Blended and Conventional

Sampling Methods and Inclusion Criteria: The overall scope of the pilot (20 blended IRT treatment group districts and 20 conventional IRT control group districts) was determined by the main research stakeholder, the MOH, as previously outlined. All learners who attended at least one training session were included in reporting. Analyses of knowledge and skills assessment data were restricted to learners who successfully completed both a pre- and post-training assessment. Blended training HEW Supervisors and IRT Course Facilitators were invited to participate in the post-training surveys.

Due to resource constraints, only a subset of blended training HEWs were invited to participate in skills assessments before and after IRT. For this group, a sample size of at least 168 was determined by using Cochran’s adjusted formula for smaller populations with the inputs of a 95% confidence level (corresponding z-score of 1.960), standard deviation of 0.5, 7% margin of error, and total estimated population size of 1172 blended learners [30]. This assessment was not conducted for the conventional IRT control group because the traditional training module did not include a skill assessment component.

Data Collection and Quality Assurance: Learners’ use of EE, including activity completion and time spent, was passively collected through the app. Blended IRT treatment group learners completed their pre- and post- knowledge assessments and surveys directly in the EE app, whereas conventional IRT control group learners completed paper-based tools administered by site coordinators (in accordance with standard practice for in-person IRT). Paper based forms were then digitally entered into ODK Collect. Facilitator surveys, supervisor surveys and skills assessments were collected via ODK and uploaded to a central server when connected to the internet [31]. Data collectors were trained on data quality checks to implement during data collection and data entry, including review of data completeness and alignment between paper-based forms and digital data entry. Data quality assurance measures were conducted prior to analysis, such as checking for missing data, removing duplicates and ensuring correct matching of learner IDs across various forms.

Quantitative Data Analysis: Data collected within the EE app and ODK Central were imported into STATA version 17.0 for cleaning, restructuring and analysis [31]. Univariate descriptive analyses were conducted to determine the reach of the RMNCH Blended IRT, including the number of learners overall, number and percentage of learners completing IRT, demographic characteristics of learners, duration and frequency of use of the EE app, knowledge, skills and satisfaction with the training (for learners, course facilitators and HEW Supervisors). Bivariate analyses were conducted to compare IRT completion and knowledge, by region, demographic characteristics and treatment group. For data collected from the blended and conventional IRT control group sites pre- and post-training, a difference-in-differences (DID) analysis was used to determine whether there was a greater change in RMNCH knowledge scores among the Blended IRT treatment group compared to the conventional IRT control group.

(Treatment_post - Treatment_pre) - (Control_post - Control_pre) = Difference-in-Difference estimate

Key covariates included geographic region (nominal), experience level (categorized as <2 years, 2–5 years, 6–10 years and 10+ years) and age (continuous), and were selected *a priori* based on their possible predictive or explanatory effects on the dependent variable of RMNCH knowledge scores. Additional covariates explored during analysis included exposure to prior in-service training (binary: no/yes) and level of education (categorized as Level III or Level IV or higher). The difference-in-differences analysis was conducted first using an unadjusted model, and then adjusting for potential covariates. *P* values were considered significant if they were less than 0.05.

Costing Data Analysis: Training expenditures data were entered into a Microsoft Excel-based tool for cost modeling and data analysis. Costing methods included a combination of activity- and ingredients-based approaches.[33] For the purposes of the cost comparison, costs were grouped according to the categories listed below:

- App development and maintenance costs and the costs of developing content for both training modalities
- User testing costs, including developing and printing ‘hard copy’ training materials for the fully in-person training modality
- Costs associated with training of facilitators on the app and the blended IRT approach prior to training implementation
- Recurrent training costs for HEWs: Costs associated with in-person training sessions in both blended and conventional IRT control group sites, including per diem, transport and fees for facilitators, training facility rental and training materials costs, per diem and transport for learners and other non-participant costs.
- Equipment costs needed for training, including costs for new and replacement tablets and chargers.
- Recurrent (ongoing) post-training costs for learners: Learners will continue to use tablets to facilitate continued learning and exploration of training materials after blended IRT is completed. This will require continued expenditure for periodic equipment replacement during deployment.

## RESULTS

### Evaluation topic #1: reach of the training pilot

Blended training was conducted with 1000 HEWs and 122 HEW Supervisors; of these, 1097 completed both a pre- and post-training knowledge assessment. Conventional IRT control group in-service training was conducted with 970 HEWs and 27 HEW Supervisors; of these, 978 completed both a pre- and post-training knowledge assessment. Additional pilot participants included 88 course facilitators, 38 health information technicians and 10 training coordinators.

The proportion of HEWs was higher at conventional IRT control group sites (97%) than blended learning sites (90%). To ensure comparability, analyses comparing the two groups were restricted to HEWs, resulting in 1000 HEWs in the blended IRT treatment group and 970 in the conventional IRT control group for the following analyses. After restricting the analysis to only HEWs, the demographics and professional experience were broadly similar for learners in the Blended IRT treatment and conventional IRT control groups, as shown in Table 3. Nearly all HEWs who participated in the training were female (100% of blended learners vs 99.9% of conventional IRT control group learners) and had similar levels of experience (51% of blended learners had 10+ years of experience vs 50% of conventional IRT control group learners). However, HEWs who received the blended training were significantly less likely to work in a rural health post (93% vs 100%, *P* < 0.001), had completed a higher level of training (71% vs 66%, *P* = 0.010), were more likely to have previously attended an RMNCH IRT (64% vs 57%, *P* = 0.002), and were slightly older (mean age of 29.8 vs 28.2, *P* < 0.001) as compared to the conventional IRT control group. Completion rates by data collection tool are provided in Supplementary Material (Appendix), Table 5.

**Table 3:** Learner demographic and professional characteristics (HEWs only).

**Learner Characteristic**	**Blended IRT** **n = 1122**	**Conventional IRT n = 997**	***P* value**
Female	100.0%	99.9%	0.310
Region: Amhara Oromia Sidama SNNP	36.5% 31.4% 12.5% 19.6%	40.0% 30.8% 12.9% 16.3%	0.196
Rural^*^	93.0%	100.0%	**<0.001**
Previously attended any IRT	65.1%	60.9%	0.060
Previously attended RMNCH IRT	64.1%	57.1%	**0.002**
Highest training: Level IV	71.2%	65.8%	**0.010**
Years experience as HEW: <2 2–5 6–10 10+	10.1% 23.7% 15.2% 51.0%	11.1% 20.6% 18.0% 50.4%	0.180
Age in years (mean)	29.8	28.2	**<0.001**

^*^Based on rural health post designation

Bold p-values indicate statistical significance at the 5% level (*P* < 0.05)

### Evaluation topic #2: blended training learner use of and reaction to extension essentials app

**Figure 1:**
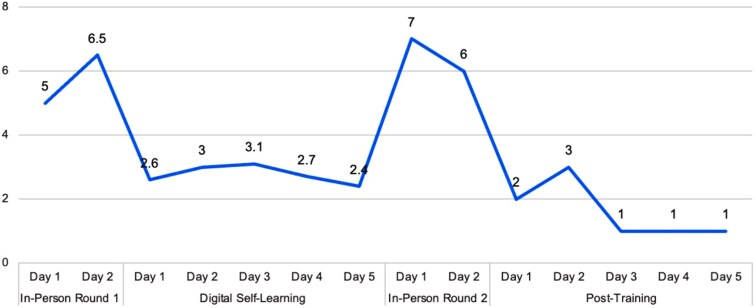
Average daily hours spent in the extension essentials (IRT) app by health extension workers during the training period (n = 997). Learners used the app for a mean of 2.8 hours per day, exceeding the 2-hour target, with peaks during in-person sessions. The x-axis shows training period (*In-person round 1, digital self-learning, In-person round 2* and *post-training*); the y-axis shows mean daily use in hours. Error bars represent 95% confidence intervals.

**Figure 2:**
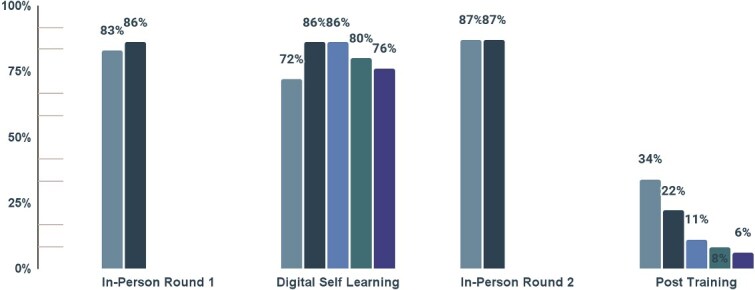
Proportion of health extension workers with daily extension essentials (IRT) app use during the training period (n = 997). App use was highest during in-person sessions (rounds 1 and 2) and remained steady during the self-learning phase, but declined post-training. The x-axis shows consecutive training days within each phase (distinguished by color), and the y-axis shows the percentage of HEWs logging in.

Learners used the app extensively during the digital self-learning period, averaging 2.8 hours per day. Based on the training schedule, practical context and self-learning activities assigned, it was estimated that 2 hours per day would be a reasonable and desired target for their engagement. Figures 1 and 2 illustrate that engaging content, a clear daily schedule, and the training design likely contributed to consistent utilization during the self-learning period. High daily usage during in-person round 1 and round 2 training sessions reflects time spent learning to use the app, participating in pre- and post-training testing and learning from digital content under the guidance of a trained facilitator.

**Figure 3:**
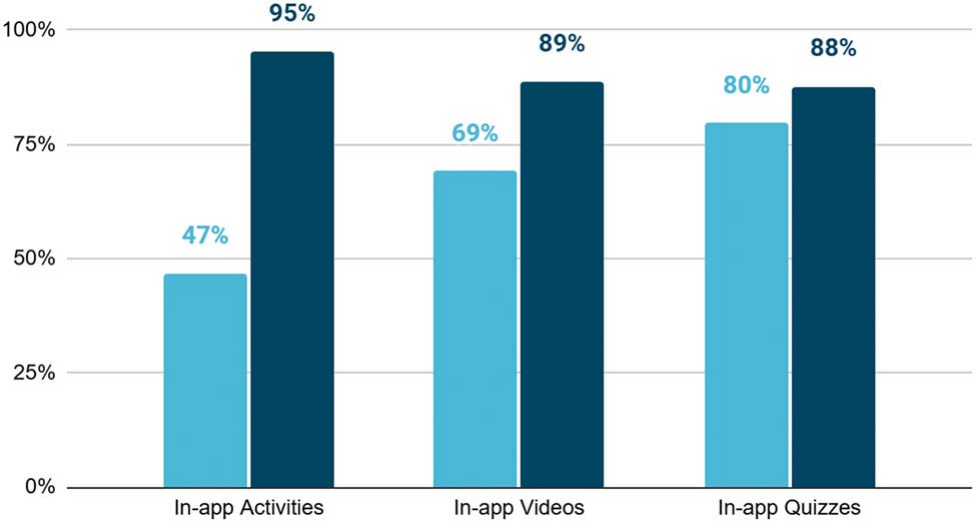
Percent of blended HEWs completing digital learning activities (n = 1000). Completion rates were highest for in-app activities (95%), followed by in-app videos (89%) and quizzes (88%). The x-axis shows activity type (*In-app activities, videos, quizzes*), and the y-axis shows the percentage of HEWs (0%–100%). The distinct bars indicate HEWs who completed all activities versus those who completed ≥80%.

Blended training participants were expected to interact with at least 80% of in-app content, a threshold set by developers to effectively absorb the information and gain familiarity with the tools. This target was communicated to learners as part of the course expectations. Nearly all blended learners surpassed completion threshold for in-app activities (95%), in-app videos (89%) and in-app quizzes (88%), demonstrating strong engagement with digital components of the blended approach (Fig. 3).

**Figure 4:**
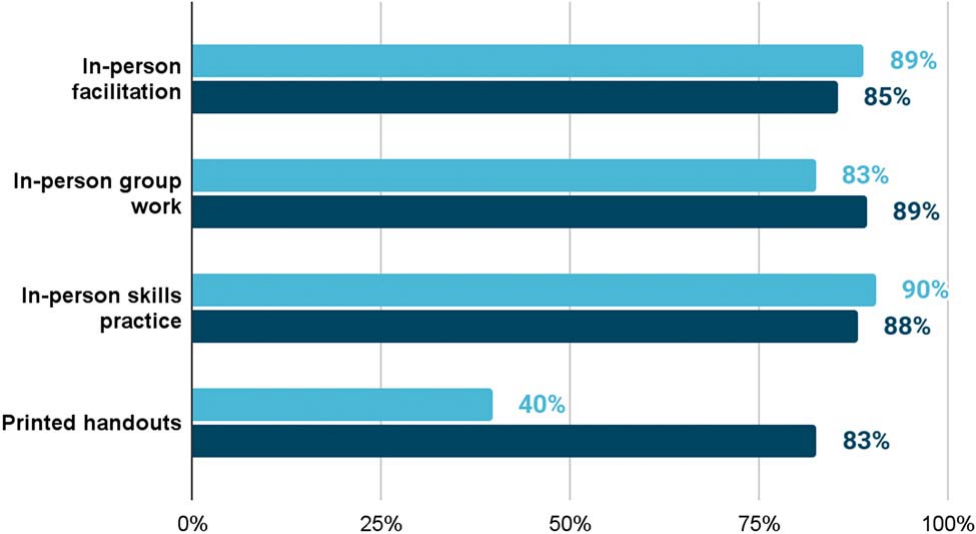
Participants’ perceptions of in-person training components (n = 997). Facilitation, group work and skills practice were rated most positively, whereas printed handouts received the lowest usefulness scores. Bars represent the proportion of participants rating each component as *‘extremely useful’* on a four-point Likert scale. The x-axis shows training components, and the y-axis shows the percentage of participants. Light blue bars indicate facilitators, and dark blue bars indicate HEWs.

**Figure 5:**
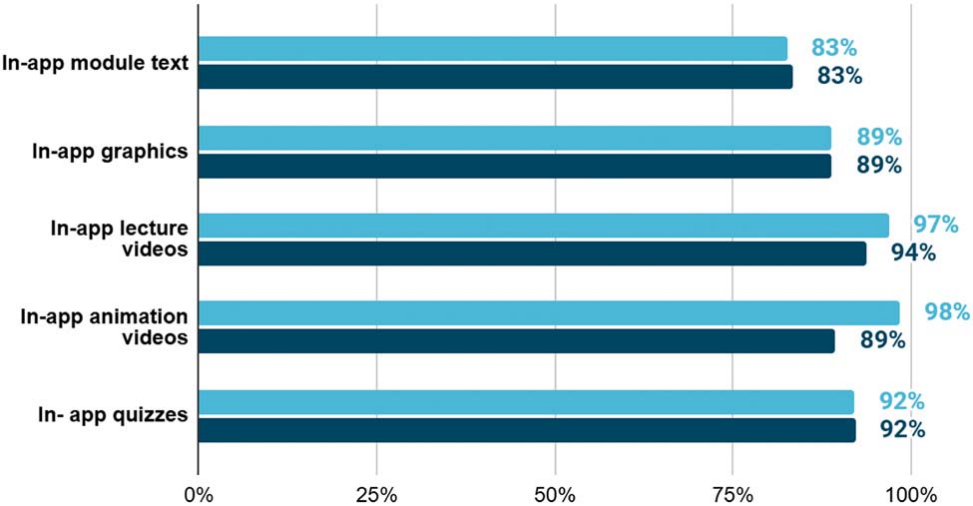
Perceived usefulness of in-app training components (n = 997). Lecture videos (94%), quizzes (92%) and animation videos (89%) received the highest proportion of ‘extremely useful’ ratings. Bars show the percentage of participants selecting ‘extremely useful’ for each digital component. X-axis shows percent of HEWs component ratings. Y-axis shows in-app content components. The distinct bars indicate whether the responses are from facilitators or HEWs.

Learners rated all in-app training components positively. Lecture videos (94%), quizzes (92%) and animation videos (89%) received the highest proportion of ‘extremely useful’ ratings (most favorable response on the four-point Likert scale), demonstrating a positive reaction to the blended components of the training. While participants also valued in-person components including facilitation, group work and skills practice, the prominent training modality used in conventional IRT (printed handouts) received the lowest usefulness score, especially as rated by facilitators (Fig. 4 and Fig. 5).

**Figure 6:**
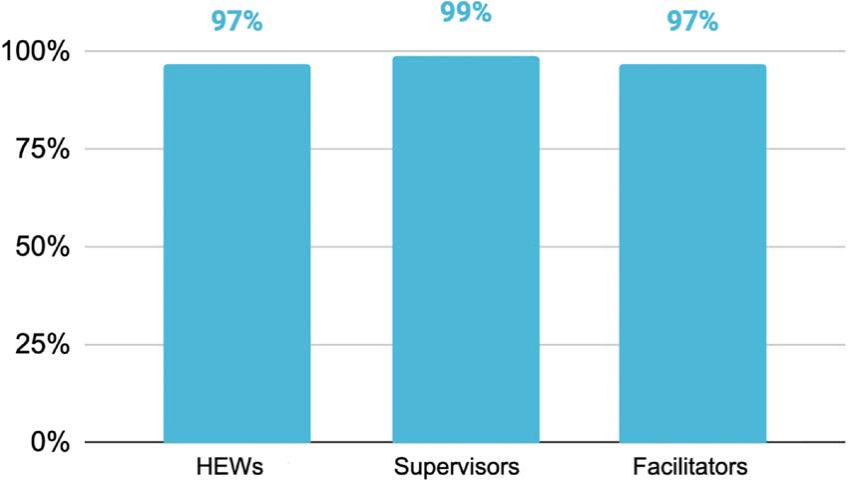
Percent of blended training participants who recommend scaling the blended approach nationally (HEWs = 978, supervisors = 121, facilitators = 63). Across participant groups, 97–99% recommended national scale-up. Bars indicate the proportion of each participant group selecting ‘recommend scaling’ in the study. The x-axis shows participant type, and the y-axis shows the percentage of participants recommending scale-up.

Participants in the blended training (HEWs, facilitators and HEW supervisors) expressed a high degree of satisfaction with the approach, with 97 to 99% of key participant groups recommending scaling the blended approach nationally (Fig. 6).

Blended IRT facilitators reacted well to the blended approach: the majority (86%) felt well-prepared, and only 8% felt it was challenging to utilize the blended approach. 100% of facilitators reported that learners were highly engaged.

### Evaluation topic #3: blended training impact on knowledge and skills in key RMNCH competencies

**Figure 7:**
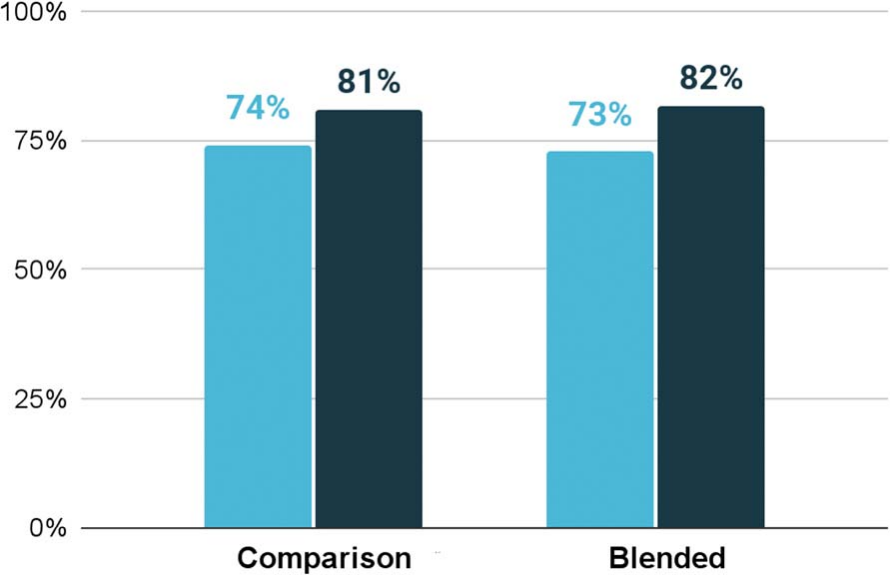
Learner knowledge assessment scores before and after training, by treatment group. Conventional IRT control group scores increased from 73.79% pre-test to 80.90% post-test, while blended IRT treatment group scores increased from 73.11% to 81.95%, reflecting improvements of 7.11 and 8.85 percentage points, respectively. Bars indicate mean scores before and after training for each group, with error bars showing 95% confidence intervals. The x-axis displays the treatment and IRT control/comparison groups at pre- and post-training, and the y-axis shows learner knowledge assessment scores. Light blue bars represent pre-training scores, and dark blue bars represent post-training scores.

Conventional IRT control group knowledge scores increased from an average of 73.79% pre-test to 80.90% post-test, an improvement of 7.11 percentage points. Blended IRT treatment group knowledge scores increased from an average of 73.11% pre-test to 81.95% post-test, an improvement of 8.85 percentage points. Figure 7 illustrates changes in learner knowledge assessment scores before and after training, disaggregated by treatment group.

An unadjusted difference-in-differences analysis showed that the 1.86 percentage point larger increase in knowledge scores among blended learners was statistically significant (*P* = 0.004; 95% CI: 0.6–3.1). The difference-in-difference analysis adjusted for key covariates of interest (age, region experience, education and prior training) yielded similar results (2.01 larger percentage point increase, *P* = 0.001; 95% CI: 0.8–3.2). While the blended learners did improve slightly more, the difference was not programmatically meaningful in terms of learner outcomes. Both groups scored similarly on pre- and post-training knowledge assessments. These findings suggest the blended approach is just as effective as conventional IRT at achieving knowledge gains.

**Figure 8:**
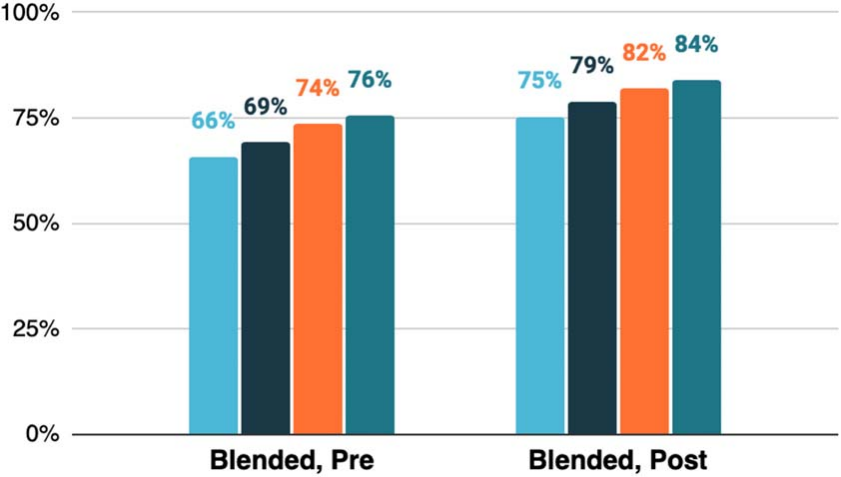
Mean knowledge assessment score, by years of experience as a health extension worker (n = 977). Blended training HEWs with more years of experience scored higher on both pre- and post-tests, with those having ≥10 years scoring an average of 8.6 points higher on the post-test than those with <2 years (*P* < 0.001; 95% CI: 6.4–10.8). Bars indicate mean scores by experience category, with error bars representing 95% confidence intervals. The x-axis shows years of experience and the y-axis shows mean knowledge assessment scores pre and post training for each experience category. The distinct bars distinguish experience categories (<2 years, 2–5 years, 6–10 years, >10 years).

Blended training HEWs with more years of work experience tended to score higher on both pre- and post-knowledge assessments, suggesting that the technological platform was not a barrier to experienced HEWs (Fig. 8). HEWs with at least 10 years of experience scored an average of 8.6 points higher on the post-test than those with less than 2 years of experience (*P* < 0.001; 95% CI: 6.4–10.8).

**Figure 9:**
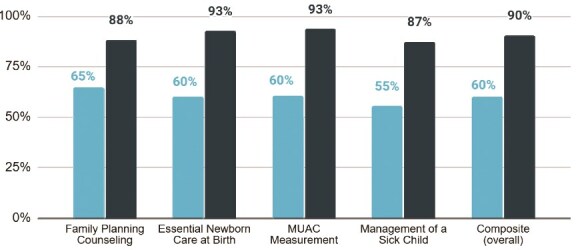
Mean skills assessment score among 174 sampled blended training learners. Scores demonstrate substantial improvement following training. Bars indicate mean skills scores, with error bars representing 95% confidence intervals. The x-axis shows different skill types, and the y-axis shows mean skills assessment scores before and after training. The distinct bars distinguish pre-training and post-training scores.

After training, skills assessment scores among blended IRT learners improved dramatically for all RMNCH key competencies. The average composite skills assessment score increased by 30 percentage points (*P* < 0.001; 95% CI: 27.4–33.2) from 60% to 90%. Skills assessments were conducted only for a sample of blended IRT treatment group learners (n = 174) and were not conducted for conventional IRT control group learners because the traditional training module did not include a skill assessment component. Skills assessments reflect learners’ ability to apply information to the practice of their job duties and demonstrate a substantial improvement in performance after training (Fig. 9) highlighting the significant performance gains following the training. Detailed analyses of pre- to post-training knowledge score changes stratified by region, age and years of experience are available in Supplementary Material (Appendix), Table 6.

### Evaluation topic #4: use of training data in follow-up supervision visits

**Figure 10:**
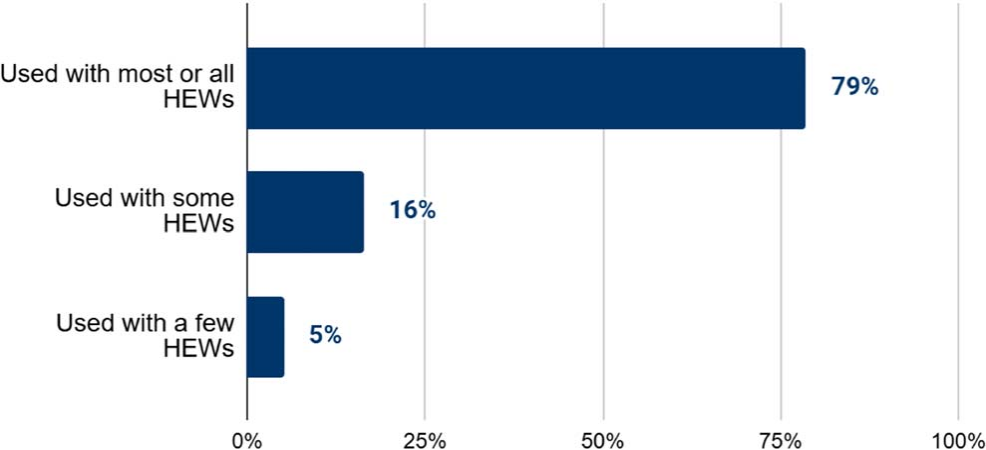
HEW supervisor use of knowledge assessment report to supervise the HEWs they oversee (n = 98 supervisors). Supervisors used the reports during routine support and follow-up to identify and address knowledge gaps in real time. Bars indicate the proportion of supervisors using the report for each monitoring activity. The x-axis shows the percentage of supervisors using the knowledge assessment report, and the y-axis shows the different ways the report was used.

All HEW supervisors received learner performance reports for their supervisees, enabling them to use the results to guide and monitor their progress. All HEW who received learner reports for reported using them in follow-up supervision visits. Of those who received a report, 98% indicated they found the reports useful, and 90% indicated that they used the report in planning and conducting supervision visits. This level of engagement demonstrates that supervisors found the reports valuable and user-friendly for monitoring HEW progress. More importantly, supervisors utilized these reports during routine support and follow-up to identify and address knowledge gaps in real-time, directly linking digital learning with practical service delivery. This is illustrated in Fig. 10, which shows HEW supervisors’ use of knowledge assessment report to supervise the HEWs they oversee.

### Evaluation topic #5: cost differences between blended (treatment) and conventional (control) training

**Figure 11:**
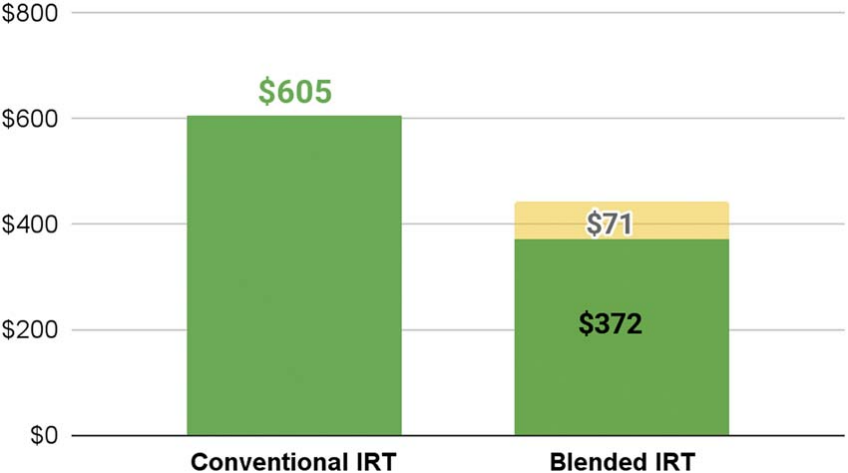
Cost per HEW trained, blended IRT vs. conventional IRT. Recurring costs for the blended training were 39% lower than the conventional approach, and total costs including one-time app development remained lower. Bars indicate the cost per HEW for each training model, with error bars showing variability across implementation sites. The distinct bars indicate recurring costs versus one-time costs (app development cost). The x-axis shows the type of training, and the y-axis shows the cost per HEW trained.

The blended IRT approach costs less than conventional IRT. Recurring costs associated with running the blended training were 39% lower than the conventional approach. Even when including one-time costs such as app development, the blended approach was still less expensive. Scaling the blended training beyond the initial 1000 HEWs included in the pilot will further distribute the fixed up-front costs associated with this model (Fig. 11).

## DISCUSSION

The pilot implementation of the EE blended learning approach demonstrates its potential to enhance the competency of HEWs in rural Ethiopia while offering a cost-effective and scalable model for in-service training. The findings align with global evidence supporting blended learning as a viable strategy for healthcare worker training in low-resource settings [12, 16].

The blended learning modality achieved comparable knowledge gains to conventional training (82% vs. 81% post-test scores) and demonstrated effectiveness in skill acquisition, with skills assessment scores increasing from 60% to 90% among blended learners. This improvement underscores the value of interactive digital components—such as animated videos, quizzes and case studies—in reinforcing practical competencies. The app’s ability to provide immediate feedback and repeated practice likely contributed to this improvement, a finding consistent with studies highlighting the role of multimedia tools in skill retention [11, 23].

Furthermore, the higher engagement with the app (2.8 hours/day) suggests that self-paced learning allows HEWs to internalize complex concepts at their own pace, which is critical for adult learners [13].

The blended approach reduced recurring training costs by 39%, primarily by minimizing expenses associated with prolonged in-person sessions, such as travel, accommodation and printed materials. These savings align with prior research indicating that blended models optimize resource allocation without compromising quality [15, 22].

Scaling the program nationally could further distribute the upfront costs of app development, enhancing cost efficiency. Notably, the high completion rates (95% for activities, 88% for quizzes) reflect the platform’s ability to deliver structured training efficiently, reducing time away from fieldwork—a critical advantage in resource-constrained settings.

The offline functionality of the EE app directly addresses connectivity barriers, enabling HEWs in remote areas to access training materials without internet dependency. This feature is particularly significant in Ethiopia, where limited digital infrastructure has historically hindered the reach of e-learning initiatives [27].

Participants’ high satisfaction with the app’s usability (e.g. local language support, intuitive design) further underscores its suitability for low-literacy populations, echoing successes of similar mHealth interventions in Sub-Saharan Africa [17, 20].

The pilot’s success in 20 districts provides a blueprint for national scalability. The app’s modular design allows for easy updates to content, ensuring alignment with evolving healthcare priorities, such as NCDs and major communicable diseases such as TB, Malaria and HIV. Supervisor reports, which 98% found useful for follow-up visits, also create a feedback loop to sustain quality improvement. However, long-term sustainability requires addressing recurrent costs, such as tablet maintenance and facilitator training. Integration of the IRT blended learning into Ethiopia’s National eHealth Strategy (2020–2025) could institutionalize funding and technical support, mitigating these challenges [27].

**Table 4:** Limitations for the RMNCH blended In-service training evaluation.

**Limitation**	**Explanation/Mitigation Strategies**
Short timeframe (7 months) and finite resources limit the opportunity to measure knowledge application or behavior change.	The study was not designed to observe changes in behavior and service delivery, as formally agreed upon with the MOH.
Limited geographic scope (40 districts out of 500+) does not capture the complex contextual information	MOH determined the geographic scope and was involved in the sampling of blended IRT treatment group districts. Analysis methods statistically control for variables that would have been controlled via study site randomization.
Research relies on self-reported data on perceived knowledge gain, self-efficacy and program satisfaction. Possible recall bias, social desirability bias.	Used validated measures in alignment with the various evaluation frameworks. Trained enumerators to emphasize the purpose of the pilot as a quality improvement exercise.
conventional IRT control group site learners self-administer paper forms vs. blended IRT treatment group site learners self-administer digital tool with data quality checks built into form design.	The intervention is housed within a digital application. It would not have been appropriate to use paper tools in the blended IRT treatment group sites. Limited resources did not allow the procurement of data collection tablets for use in the conventional IRT control group sites.
Skills assessments were only administered in the blended IRT treatment group sites and scoring by facilitators may introduce variation or bias.	Skills assessments were conducted at skills labs within CPD centers; however, conventional trainings did not occur at CPD centers. Facilitators were trained on a validated rubric for scoring the skills assessments and oriented to the purpose of the exercise as a learning opportunity, rather than a judgment of their facilitation skills.
Selection differences between the blended IRT treatment group and conventional IRT control group before the pilot is implemented could account for observed differences in outcomes between the blended IRT treatment group and conventional IRT control group.	blended IRT treatment group and conventional IRT control group sites were matched on key characteristics including location, population, health care infrastructure, health workforce, overall performance and exposure to digital health interventions. Difference-in-differences (DID) statistical analysis conducted to estimate causal effects when treatment assignment is non-random.
Testing: Taking the same post-test as had been administered as a pre-test can produce higher post-test scores due to gaining familiarity with the testing procedure.	The pre/post needs to align with country-wide assessment of HEWs for certification purposes. It was not appropriate for the study team to alter this assessment, therefore this threat to internal validity will need to be accepted. Additionally, this issue is present in equal measure in both the blended IRT treatment group and conventional IRT control groups.

### Limitations and future directions

This study has several limitations that should be considered when interpreting the findings. First, the short timeframe (7 months) and resource constraints limited our ability to assess long-term outcomes such as sustained knowledge retention, behavior change and impact on service delivery. Second, the study was implemented in a limited number of districts (40 out of 500+ nationally), which may not fully capture the diversity of geographic and contextual factors across Ethiopia.

In addition, skills assessments were conducted only among a sample of blended training participants due to resource constraints and were not included in the conventional training control group training module. This limits direct comparison of skill outcomes between groups. Learner data were primarily self-reported, which may introduce recall and social desirability biases. Furthermore, learners in blended IRT treatment group sites completed digital assessments with built-in data quality checks, while conventional IRT control group site learners completed paper-based assessments, introducing potential variation in data quality.

Despite these limitations, the study used matched conventional IRT control group districts and applied difference-in-differences analysis with adjustment for key covariates to strengthen internal validity. Detailed mitigation strategies are presented in Table 4.

Future research should explore the longitudinal impact of the blended learning model on HEW performance and service delivery outcomes. Additionally, evaluation of the model’s application in other training modules—such as those on NCDs and communicable diseases—would help assess its scalability and adaptability across Ethiopia’s health system.

## CONCLUSION

The EE blended learning model offers a pragmatic solution to Ethiopia’s community healthcare training challenges. By combining cost savings, skill enhancement and offline accessibility, it aligns with global calls for innovative, scalable health workforce strategies [8, 20]. Policymakers should prioritize its integration into national training frameworks while investing in infrastructure to ensure equitable access across all regions.

## Supplementary Material

Supplementary_material_Appendix_Publication_abstract_app_oqaf022

## Data Availability

The data underlying this study are not publicly available. However, the data can be made available by the corresponding author upon reasonable request.
