# Supplementary material for: Empowering community health workers in rural Ethiopia with blended learning: an offline mobile application to enhance trainings and healthcare services
Source: Oxf Open Digit Health. 2025 Sep 9;3:oqaf022. doi: 10.1093/oodh/oqaf022 (PMC12461708; doi:10.1093/oodh/oqaf022)
Supplement: Supplementary_material_Appendix_Publication_abstract_app_oqaf022 [file supplementary_material_appendix_publication_abstract_app_oqaf022.docx]

# Supplementary Materials

# Title: Empowering Community Health Workers in Rural Ethiopia with Blended Learning: An Offline Mobile Application to Enhance Trainings and Healthcare Services

## Authors and Affiliations:

Asmamaw, Etsegent Arega, Ethiopia Country Program, Last Mile Health, Addis Ababa, Ethiopia, easmamaw@lastmilehealth.org

Begley, Mark Ryan, Global MERL, Last Mile Health, Syracuse, NY, USA, [mbegley@lastmilehealth.org](mailto:mbegley@lastmilehealth.org)

Bele,Temesgen Ayehu, Ethiopia Country Program, Last Mile Health, Addis Ababa, Ethiopia, [temesgenayehu0@gmail.com](mailto:temesgenayehu0@gmail.com)

Debar, Ruth Diriba, Program, Ethiopia Country Program, Last Mile Health, Addis Ababa, Ethiopia, [rdiriba@lastmilhealth.org](mailto:rdiriba@lastmilhealth.org),

Egnuni, Tamene Feyissa, Ethiopia Country Program, Last Mile Health, Addis Ababa, Ethiopia, [tfeyissa@lastmilehealth.org](mailto:tfeyissa@lastmilehealth.org),

Krause, Julie Anne, MERL, Ethiopia Country Program, Last Mile Health, Cherry Hill, NJ, USA, [jkrause@lastmilehealth.org](mailto:jkrause@lastmilehealth.org)

Megenetta, Abraham Zerihun, Ethiopia Country Program, Last Mile Health, Addis Ababa, Ethiopia, [azerihun@lastmilehealth.org](mailto:azerihun@lastmilehealth.org)

Otoro, Israel Ataro, Primary Health Care and Community Engagement Lead Executive Office, Ministry of Health, Addis Ababa, Ethiopia, [iarelotoro1@gmail.com](mailto:iarelotoro1@gmail.com),

Sado**,**Tibebu Benyam, Ethiopia Country Program, Last Mile Health, Addis Ababa, Ethiopia, tbenyam@lastmilehealth.org

Wendmagegn, Netsanet Fetene, Ethiopia Country Program, Last Mile Health, Addis Ababa, Ethiopia, [nfetene@lastmilehealth.org](mailto:nfetene@lastmilehealth.org)

**Corresponding Author**
Egnuni, Tamene Feyissa, Ethiopia Country Program, Last Mile Health, AG Grace Building, 8th Floor, Djibouti Road, Bole Sub-city, Addis Ababa, Ethiopia,
tfeyissa@lastmilehealth.org

## Appendix

Blended RMNCH In-Service Training Data Collection Tool Completion Rates

Table 5: Blended RMNCH In-Service Training completion rates by data collection tool

| **Data Collection Tool** | **Conventional in-service training** | **Blended in-service training** |
| --- | --- | --- |
| **Knowledge Assessment** | 98% HEW (952/970)  100% HEW Supervisor (26/27) | 98% HEW (978/1,000)  98% HEW Supervisor (119/122) |
| **Learner Survey: Background Characteristics** | 100% HEW (970/970)  100% HEW Supervisor (27/27) | 99% HEW (992/1,000)  97% HEW Supervisor (118/122) |
| **Learner Survey: Digital Tools Comfort/Experience** | 100% HEW (970/970)  100% HEW Supervisor (27/27) | 99% HEW (992/1,000)  99% HEW Supervisor (121/122) |
| **Skills Assessment** | N/A | 96% HEW *sample* (174/181) |
| **Supervisor Survey** | N/A | 91% HEW Supervisor (111/122) |
| **Facilitator Survey** | N/A | 82% Facilitator (73/89) |
| **Learner Focus Group Discussions** | N/A | 90% HEW *sample* (145/162) |

For knowledge and skills assessments, completion is defined as completing both pre- and post-training assessments. An additional 35 conventional IRT control group site learners completed only post-training learner surveys. Because learner characteristics were captured on pre-training surveys, this information is not available for this group, which was excluded from analysis.

Blended RMNCH In-Service Training Knowledge Assessment Score Detailed Analysis

Table 6: Blended RMNCH In-Service Training pre- to post-training change in knowledge assessment scores among HEWs, stratified by key covariates

|  | Blended IRT treatment group | | | Conventional IRT control group | | |
| --- | --- | --- | --- | --- | --- | --- |
|  | Pre- training score | Post- training score | Mean change,  pctg. pts.  (95% CI) | Pre- training score | Post- training score | Mean change,  pctg. pts.  (95% CI) |
| Region |  |  |  |  |  |  |
| Oromia | 74.4% | 83.2% | +8.7* (7.8, 9.7) | 71.7% | 79.0% | +7.3* (6.2, 8.3) |
| SNNP | 67.6% | 76.5% | +8.9* (7.5, 10.4) | 72.1% | 81.9% | +9.8* (8.0, 11.5) |
| Sidama | 67.7% | 79.8% | +12.1* (9.9, 14.3) | 72.1% | 78.5% | +6.6* (4.9, 8.4) |
| Amhara | 75.8% | 83.5% | +7.7* (6.9, 8.5) | 76.8% | 82.4% | +5.7* (5.0, 6.4) |
| Age |  |  |  |  |  |  |
| 20-24 years | 72.0% | 79.9% | +7.9* (6.3, 9.5) | 71.8% | 79.6% | +8.0* (6.6, 9.3) |
| 25-29 years | 71.1% | 80.2% | +9.1* (8.1, 10.1) | 73.4% | 80.6% | +7.3* (6.4, 8.1) |
| 30-34 years | 74.4% | 83.2% | +8.9* (7.9, 9.8) | 75.3% | 81.8% | +6.5* (5.4, 7.6) |
| 35-39 years | 73.8% | 82.6% | +8.8* (7.3, 10.3) | 77.0% | 81.7% | +4.7* (3.2, 6.2) |
| 40+ years | 73.8% | 82.4% | +8.6* (5.4, 11.7) | 67.8% | 75.8% | +8.0* (4.5, 11.6) |
| Years of Experience | | |  |  |  |  |
| <2 years | 65.7% | 75.3% | +9.6* (7.1, 12.1) | 69.0% | 79.0% | +10.2* (8.2, 12.1) |
| 2-5 years | 69.2% | 78.9% | +9.7* (8.4, 11.1) | 72.4% | 79.2% | +6.9* (5.7, 8.1) |
| 6-10 years | 73.5% | 81.9% | +8.4* (7.0, 9.8) | 73.9% | 81.0% | +7.2* (5.9, 8.5) |
| >10 years | 75.6% | 84.0% | +8.3* (7.6, 9.1) | 75.5% | 81.7% | +6.2* (5.4, 7.0) |

Analysis of variance testing indicated there was statistically significant variation (p<.05) in mean pre- to post-training knowledge score change among blended learners when stratified by region, and among conventional IRT control group when stratified by region, age, or years of experience. Note that mean change may appear not to align precisely with associated pre- and post-training scores due to rounding.
